# Effectiveness of Bobath Therapy vs. Conventional Medical Gymnastics in Psycho-Social and Cognitive Status Improvement in Children with Mild Neurodevelopmental Delay: A Randomized Double-Blinded Prospective Cohort Study

**DOI:** 10.3390/biomedicines12122767

**Published:** 2024-12-05

**Authors:** Zrinka Djukić Koroljević, Luka Bulić, Eva Brenner, Matea Bračić, Petar Brlek, Dragan Primorac

**Affiliations:** 1 St. Catherine Specialty Hospital, 10000 Zagreb, Croatialuka.bulic0302@gmail.com (L.B.);; 2 Department of Neurology, University Hospital Center “Sisters of Mercy”, 10000 Zagreb, Croatia; 3 Medical School, Josip Juraj Strossmayer University of Osijek, 31000 Osijek, Croatia; 4 Department of Molecular Biology, Faculty of Science, University of Zagreb, 10000 Zagreb, Croatia; 5 Eberly College of Science, The Pennsylvania State University, State College, PA 16802, USA; 6 Medical School, University of Split, 21000 Split, Croatia; 7 The Henry C. Lee College of Criminal Justice and Forensic Sciences, University of New Haven, New Haven, CT 06516, USA; 8 REGIOMED KLINIKEN, 96450 Coburg, Germany; 9 Medical School, University of Rijeka, 51000 Rijeka, Croatia; 10 Faculty of Dental Medicine and Health, Josip Juraj Strossmayer University of Osijek, 31000 Osijek, Croatia; 11 Medical School, University of Mostar, 88000 Mostar, Bosnia and Herzegovina; 12 National Forensic Sciences University, Gandhinagar 382007, India

**Keywords:** neurodevelopmental disorder, habilitation, Bobath therapy, conventional medical gymnastics

## Abstract

**Background/Objectives:** The main objective of this research was to compare the Bobath concept and conventional medical gymnastics in psycho-social and cognitive habilitation of infants with mild neurodevelopmental delay, and determine whether there is a difference in their effectiveness. **Methods:** The study included 100 children up to 3 months of age who were diagnosed with a mild neuromotor disorder based on clinical examination, the Münich Functional Developmental Diagnostic Test for the first year of life, and the Ages and Stages questionnaire. The respondents were randomized into two groups, habilitated according to the Bobath concept or conventional medical gymnastics. The observed parameters were problem-solving skills, communication skills, and the infants’ psycho-social status. **Results:** The Ages and Stages questionnaire revealed no significant differences between the two concepts. The Munich Diagnostic Test revealed different starting values in speech and socialization delay, but the treatment outcomes showed significant improvement in both cohorts. There were also no significant differences in the guardians’ opinions on therapeutic effectiveness. **Conclusions:** This study demonstrated that there is no difference in effectiveness between the two tested habilitation programs in mild neurodevelopmental delay treatment in infants after six months of therapy, laying the foundation for a professional consensus.

## 1. Introduction

Neurodevelopment encompasses the development of motor skills, speech, behavioral patterns, learning, and communication. It begins in the embryonic stage and intensely progresses during the entire intrauterine period, as well as the first twelve months of postnatal life [1]. Neurodevelopment is influenced by many factors, from the expression of certain genes to environmental variables. Interaction between these factors is key for proper development, and any variance can potentially lead to noticeable deviations [2]. A deviation in the neurodevelopmental process can be classified as mild or severe [3,4]. Severe deviations include specific pathologies such as epilepsy, cerebral palsy, visual and auditory impairment, and mental retardation. On the other hand, mild disorders include delays in gross and fine motor skills, motor clumsiness, and cognitive, emotional, and psycho-social development abnormalities.

This study encompasses the two most commonly used habilitation concepts in the context of neuromotor developmental impairment: conventional medical gymnastics and the Bobath method. A habilitation concept refers to a therapeutic framework designed for functional habilitation.

Conventional medical gymnastics involves exercises in which movements are carried out without active participation from the child, implying passive exercises in which the movement is facilitated by the therapist. The main aim of conventional medical gymnastics is the improvement in the motility, endurance, strength, and flexibility of certain muscles or muscle groups, as well as posture improvement [5]. Conventional medical gymnastics is an older habilitation concept that has been proven to be effective in children with different neurodevelopmental disorders. Examples of such conditions include cerebral palsy and perinatal lesions of the central nervous system [6,7,8].

Bobath therapy is a newer concept based on motor plasticity and functional learning. This implies an individual and interactive approach focusing on motor function quality, unlike conventional methods focusing on quantitative measures. Today, it is one of the most frequently used neurohabilitation concepts [9]. It is based on learning by integrating sensory and motor signals, with facilitation as its key aspect. By correctly performing certain movements, sensory input is provided, which leads to better motor control as the brain learns based on these inputs [10,11,12]. Much like conventional medical gymnastics, the Bobath concept has also been proven to be effective in different clinical scenarios but predominantly in the case of cerebral palsy [13,14,15]. While the Bobath concept is a more expensive therapeutic approach, cost-effectiveness analyses have shown positive results. Geng et al. analyzed the cost-effectiveness of Bobath vs. traditional rehabilitation in post-stroke syndrome and concluded that Bobath was more cost-effective [16]. Additionally, a systematic review by Diaz-Arribas MJ et al. investigated the effectiveness of Bobath therapy in post-stroke rehabilitation [17]. While no significant superiority was noted regarding the mobility or motor control of the lower limb, evidence was found for superior results regarding upper limb motor control and dexterity. On the other hand, Dorsch S. et al. provided an opposing view in their systematic review [18].

While previous studies have investigated these concepts individually, a direct comparison of the effectiveness of the two concepts on this population and pathology has never been published in the literature. The aim of this study was to compare the two most commonly used therapeutic methods, conventional medical gymnastics and the Bobath concept, and determine whether there is a statistical difference in the outcomes between the two regarding psycho-social, emotional, and cognitive development. Secondly, this study aimed to demonstrate the importance of early abnormality detection and prompt intervention. Our first hypothesis was that the Bobath treatment would result in statistically significant improvements in the measured outcomes in our cohort. Our second hypothesis was that the conventional medical gymnastics treatment would result in statistically significant improvements in the measured outcomes in our cohort. Finally, our third hypothesis was that no statistically significant difference in effectiveness would exist between these two concepts.

## 2. Materials and Methods

### 2.1. Participants and Study Design

**Figure 1:**
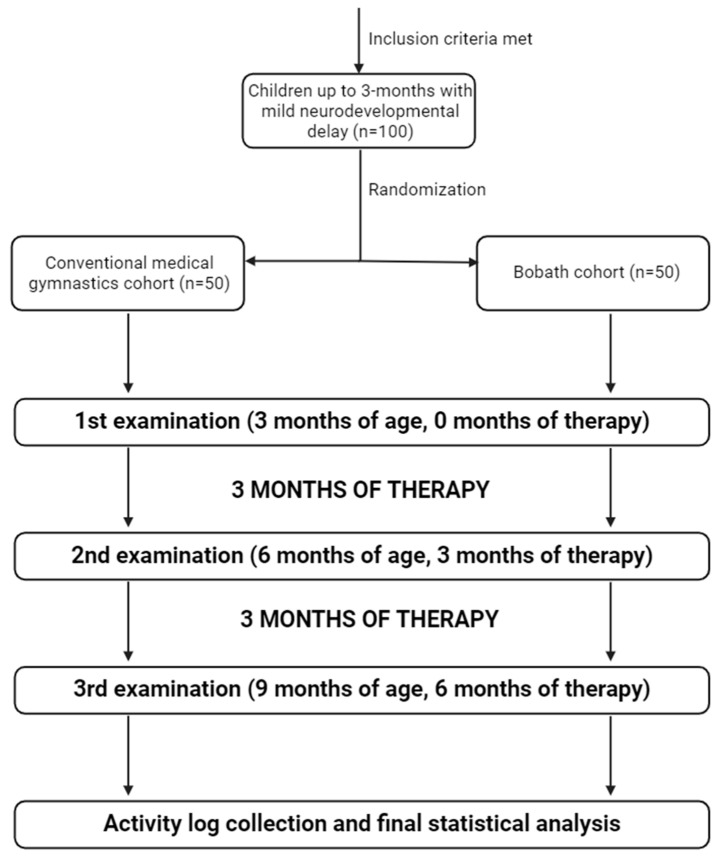
Study flow chart demonstrating the selection and treatment process of involved patients. After randomization, the selected patients were subject to three examination checkpoints: initially, after three months of therapy, and after six months of therapy (created with Biorender.com).

By design, this was a double-blinded prospective cohort study in which the legal guardian, evaluator, and statistician were unaware of the distribution of children among groups. The study included 100 children up to three months of age and of Caucasian ethnicity who had demonstrated certain deviations in their neuromotor development. The inclusion criteria were neurodevelopmental deviation of normal age-specific development diagnosed by a physiatrist, abnormal brain ultrasound findings, and positive risk factors. Risk factors included advanced maternal age, hypothyroidism, gestational diabetes, multiples pregnancy, placenta previa, IUGR, EPH gestosis, IVF-based conception, infections, Rh incompatibility, premature birth, C-section, encephalopathy in the early neonatal period, postnatal episodes of apnea or convulsions, and positive family history for neurological conditions. The exclusion criteria were any previous enrolments in a habilitation program and an age greater than three months. The children were randomly assigned to either the Bobath or conventional gymnastics group, creating two cohorts with 50 children in each (Figure 1).

### 2.2. Measures and Procedures

Assessment of neuromotor development was conducted by clinical examination performed by a specialist physiatrist, as well as using the Munich Functional Developmental Diagnostic Scale, Ages and Stages questionnaire, and multiple questionnaires filled out by the child’s legal guardian. Children who met the inclusion criteria were examined at the beginning of the study, at the age of three months, and at the age of six months; after three months of therapy, the first assessment was conducted, which included a clinical examination and the first questionnaire review. After completion of the study, the final assessment of the observed parameters occurred at nine months of age. Multiple questionnaires for keeping track of sessions were analyzed after 3 months of therapy and at the end of the study.

### 2.3. Intervention

Both groups had one hour per week of habilitation therapy sessions and speech therapy and educational rehabilitation therapy twice per month. It was recommended to exercise with the legal guardian every day for 2 h, divided into several sessions depending on the child’s daily activities. Prior to including the child in the study, the assigned therapist taught the guardian how to properly perform the exercises. Consistency in performing domicile therapy was achieved by daily phone reminders from the therapist, with the aim of reminding the guardians to perform the exercises and answering any potential questions.

### 2.4. Statistical Analysis

Statistical analysis included descriptive statistics and comparative statistics between the two cohorts. The normality of the data was assessed by the Kolmogorov–Smirnov test. Distribution differences for non-normal continuous variables were tested using the Mann–Whitney U test, Wilcoxon signed-rank test, and Friedman test. Fisher’s exact test was used to analyze categorical variables. A *p*-value less than 0.05 was considered statistically significant. The analyses were completed using the MedCalc Statistical Software (v20.112).

## 3. Results

The initial analysis of the comorbidities and risk factors showed no significant differences between the conventional medical gymnastics and Bobath cohorts (Table 1).

The neurodevelopmental status of the children was assessed at three, six, and nine months of age at three levels: communication skills, problem-solving skills, and psycho-social status (Table 2).

There was no statistically significant difference noted between the groups in any of the observed categories at any of the points of assessment.

The legal guardians were asked to evaluate their child’s developmental improvement using our questionnaire. The answers and results were compared between the groups (Table 3). The first question focused on improving psycho-social interactions, and the second question assessed the guardians’ opinions regarding the reasons for the improvement if an improvement was present.

No statistically significant difference was noted between the groups regarding any of the questions.

The participants’ activity logs were analyzed to gain better insight into the characteristics of the daily domicile sessions (Table 4). The observed parameters were the number of days exercised, the number of weekend days exercised, the length of a single series, the number of series per day, and the number of days to the first noticeable improvement.

**Figure 2:**
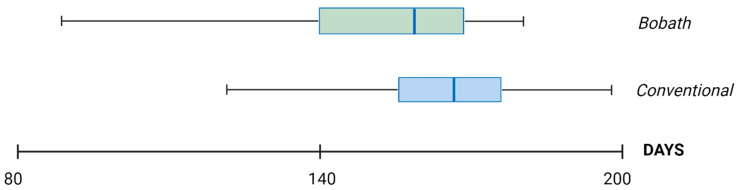
Comparison of total exercise days showing a significantly greater number of exercise days in the conventional medical gymnastics cohort (*p* = 0.012) (created with Biorender.com).

A statistically significant difference was noted in the total number of days exercised (*p* = 0.012) (Figure 2).

A box plot analysis of the distributions regarding the total exercise days showed higher values in the conventional medical gymnastics group. The other variables analyzed from the activity logs showed no statistically significant differences.

Finally, the results of the Munich Functional Developmental Diagnostic Scale were analyzed. The observed categories included perception age delay, speech age delay, and socialization age delay. The values in the table represent the number of days the child deviated in achieving a milestone from the expected time of achievement for the age-specific milestone. Initially, the improvement in delay was assessed for both cohorts in each of the observed categories (Table 5).

**Figure 3:**
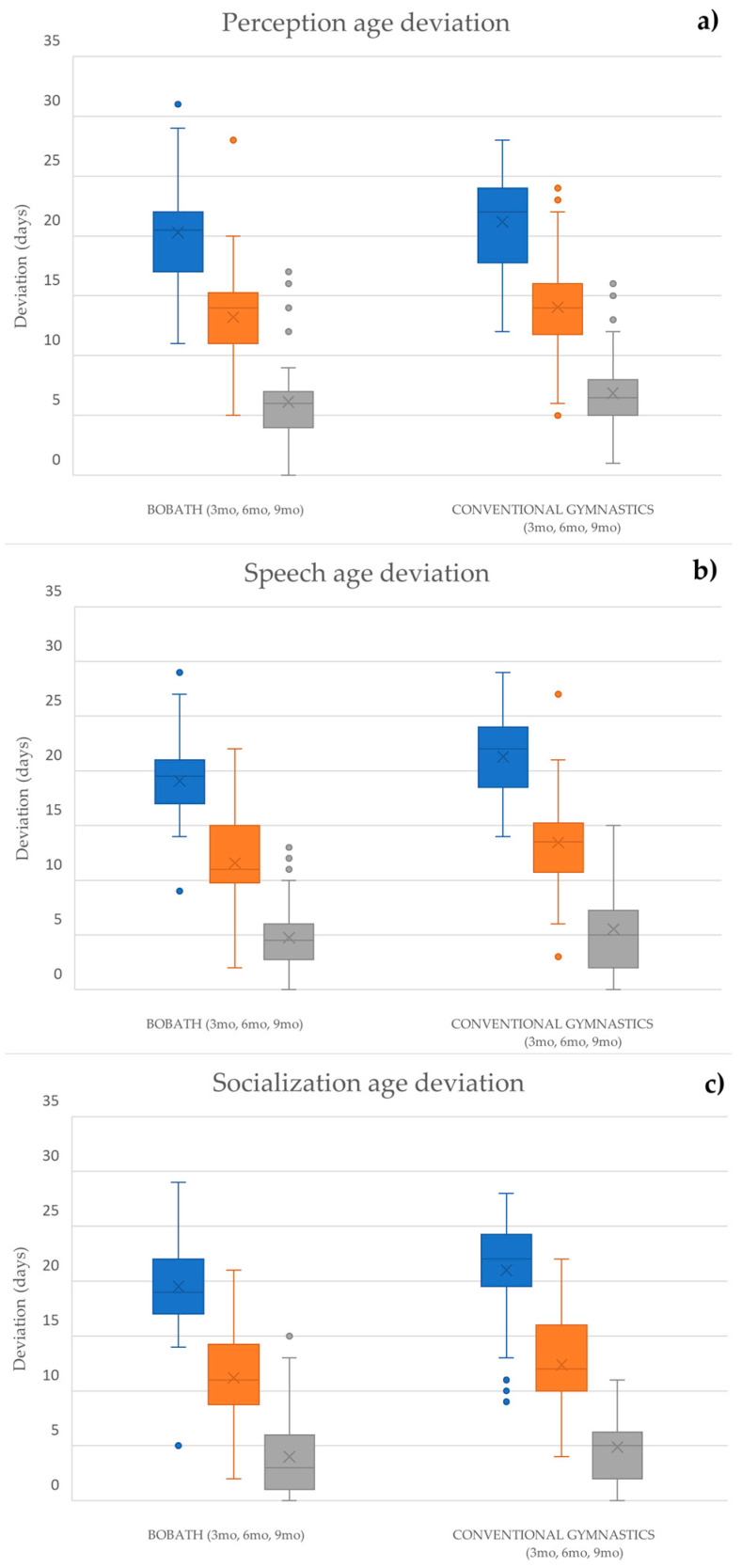
Comparison of (**a**) perception age delay, (**b**) speech age delay, and (**c**) socialization age delay trends at 3 months (blue), 6 months (orange), and 9 months (grey) of age. Both cohorts demonstrated significant improvement trends, as shown by the Friedman test (*p* < 0.001) and Wilcoxon signed-rank test (*p* < 0.001).

A significant improvement was observed in both cohorts between all three evaluation stages in all three observed categories (Figure 3).

Additionally, a statistical comparison of the scores was conducted between each of the three time points to provide a more detailed analysis of the improvement trend (Table 6).

Significant differences were noted between each of the three time points in all three categories in both cohorts (*p* < 0.001).

Secondly, a statistical comparison was conducted between the two groups to assess any differences between the two cohorts at each stage of the study (Table 7).

Statistically significant differences were noted in the speech age delay at the initial examination (*p* = 0.002) and after three months of therapy (*p* = 0.030). The conventional medical gymnastics group had greater deviations initially and after 3 months of therapy. Additionally, statistically significant differences were noted regarding the socialization age delay at the initial examination (*p* = 0.019) and after six months of therapy (*p* = 0.042). The conventional medical gymnastics cohort had greater deviations initially and after six months of therapy.

## 4. Discussion

One-hundred children, up to three months of age, were randomly assigned to the Bobath and conventional gymnastics groups, which both showed significant improvements in all the observed variables. Studies analyzing the effectiveness of these concepts alone have demonstrated significant improvements in motor skills in children with cerebral palsy following the Bobath method, as well as in children with Down syndrome following the conventional method [19,20]. In our study, similar trends were observed regarding communication skills, problem-solving abilities, and psycho-social status. However, no literature has been published regarding these categories when it comes to comparisons between the Bobath and conventional medical gymnastics concepts. In our study, the results measured by the Ages and Stages questionnaire showed no statistically significant differences initially, after three months, or after six months of therapy. There was also no statistically significant difference regarding the guardians’ opinions on therapy effectiveness. The activity log showed a significant difference regarding the distribution of total exercise days. Finally, the results of the Munich Functional Developmental Diagnostic Scale showed a statistically significant difference when analyzing speech age delay and socialization age delay initially, speech age delay after three months of therapy, and socialization age delay after 6 months of therapy.

The subjective evaluation of therapy effectiveness was based on questionnaires reporting noticeable improvements in developmental patterns. The majority of the legal guardians of the children in both groups answered that there was a noticeable positive change in social contact and interactions. Once again, there were no statistically significant differences in subjective perception regarding the effectiveness of the two methods (Table 2). Additionally, the guardians noted after how many days they noticed the first signs of improvement. The median for both groups was 30 days, and there was no statistically significant difference (Table 3). The statistical analysis of the activity logs demonstrated a statistically significant difference in total exercise days between the groups, with the Bobath group having fewer than the conventional gymnastics group. Even though the therapeutic effect achieved in both groups was equally effective, the number of days required to achieve the maximum effect differed between the Bobath group and medical gymnastics group (the effect was achieved statistically significantly faster with Bobath therapy).

The statistical analysis of the Munich Functional Developmental Diagnostics Scale results demonstrated strong therapeutic effectiveness. The longitudinal comparison of effectiveness showed statistically significant improvement trends for both cohorts. When comparing the developmental deviations in the cohorts at each stage cross-sectionally, significant differences were noted in the categories of speech age deviation and socialization age deviation. In the speech age deviation category, the conventional gymnastics group had greater initial deviation values. While this discrepancy persisted after the first three months of therapy, there was no significant difference between the groups at the end of the study. In the socialization age deviation category, the conventional gymnastics group once again had greater initial deviation values. This was the only category in which a statistically significant difference was noted at the end of the study, after six months of therapy.

The results of this study confirm that there is no statistically significant difference between the effectiveness of the conventional medical gymnastics concept and Bobath concept. According to the Ages and Stages questionnaire, both conventional medical gymnastics and Bobath therapy achieved deviation correction after six months of therapy. The instructed course of exercises for domicile therapy, which entailed three daily exercises of 30–45 min, proved to be effective and led to the normalization of psycho-social and cognitive status, as well as problem-solving abilities after six months of therapy. The strength of this research lies in the fact that these two habilitation concepts have never previously been compared regarding a cohort of children with mild developmental delay. However, other research related to these concepts has been published.

Zanon MA et al. compared the effectiveness of these two concepts on a subpopulation of children with cerebral palsy up to 18 years of age [15]. The observed outcome was an improvement in muscle strength, and the statistical analysis showed no difference between the two approaches, with no analysis of psycho-social, cognitive, and emotional development. Umber F et al. compared two cohorts, one that was treated via a combination of the Bobath and conventional medical gymnastics and one that was treated solely via the conventional method [21]. The Functional Independence Measurement Scale was used to assess the improvement of the children. The authors concluded that the addition of the Bobath method had no significant impact on the treatment outcome. However, the study by Jamil M et al. noted a certain superiority of the Bobath method [22]. Some studies analyzed the effectiveness of a combined approach of Bobath and Vojta concepts. Ungureanu A et al. assessed this approach regarding a cohort of children with cerebral palsy [23]. As shown by the Berg scale score improvements the authors used as an outcome measure, the combination of the two approaches was shown to be useful. Parau D et al. also used a combined approach with Bobath and Vojta concepts for the treatment of infants with motor development impairment [24]. The results demonstrated that the combined approach with these two concepts shortens improvement time when compared to the individual approaches. The sole Bobath approach was also tested on a cerebral palsy cohort in a study by Salatenko I et al. [25]. Once again, the result demonstrated that Bobath therapy is an effective method in cerebral palsy habilitation. Bukhovets BO and Romanchuk AP assessed the effectiveness of Bobath therapy in the correction of psychomotor development in children with injuries of the central nervous system [26]. The results demonstrated that the Bobath concept has utility in these cases as well. Some studies have explored task-specific training approaches in habilitative therapy in children with neurodevelopmental delay. One such example is locomotor treadmill training, which was analyzed in a study by Valentin-Gudiol et al. [27]. Following an extensive review, the authors found evidence that this method is also effective in subpopulations such as Down syndrome, cerebral palsy, as well as in general developmental delay. Our study also demonstrated the Bobath concept as an effective therapeutic option, in our case in infants with mild neurodevelopmental delay. The effectiveness of Bobath demonstrated in this study is in line with the published literature. However, the fact that no statistically significant difference was found between conventional medical gymnastics and Bobath suggests that conventional medical gymnastics is also worth considering as a strong therapeutic option.

The relevance of habilitation concept research in the infant population is highlighted by the frequency of neurodevelopmental disorders in infants. Namazzi et al. conducted a population-based study to investigate the prevalence and associated factors of neurodevelopmental disorders among infants [28]. In the investigated cohort of infants, the authors found that 12.7% displayed a certain neurodevelopmental deviation, while the main associated factors were multiparous pregnancy, failure to cry at birth, and post-neonatal complications. Additionally, Sanchez CE et al. demonstrated pre-pregnancy obesity of the mother as a contributing factor to neurodevelopmental deviations in the child. Considering the increasing prevalence of obesity among the general population and women of childbearing age, this presents another potential cause of the increased incidence of neurodevelopmental disorders [29].

The main limitation of this study is the fact that both conventional medical gymnastics and Bobath treatments were carried out by the children’s legal guardians. This leaves little room for control over the quality of the treatment performed, as well as whether the guardians are adhering to the prescribed protocol. This was controlled to the best of our ability with thorough education and daily reminders. All the other variables were strictly controlled and taken into account in the analysis. Another limitation of our study is the sample size. Conducting further research with a larger sample size would enhance the confidence in our findings, which were derived from a relatively small group of study participants. This limitation could be addressed by expanding the sample size, which would strengthen the validity of our conclusions. Finally, each of the evaluated concepts has its own limitations that should be addressed. For example, a meta-analysis by Te Velde A et al. demonstrated little evidence for the effectiveness of the Bobath concept in children with cerebral palsy [30]. Moreover, the authors demonstrated that a higher regimen of Bobath therapy is no more effective than a lower regimen. Similarly, Das SP and Ganesh GS mentioned the inconsistent effectiveness of physiotherapy in the cerebral palsy subpopulation [31].

## 5. Conclusions

In conclusion, the results of this study demonstrated no significant differences in effectiveness between the conventional medical gymnastics and Bobath concepts in psycho-social and cognitive status improvement in 3-month-old children with mild neurodevelopmental disorders. Under the assumption of timely treatment initiation, this suggests that the more dated and economically advantageous conventional medical gymnastics concept can lead to equally beneficial outcomes as the Bobath method. These findings might lay the foundation for building a consensus in habilitation concepts among physiatrists, leading to economically advantageous treatments and greater accessibility. Additionally, it emphasizes the importance of the early initiation of habilitation therapy.

## Figures and Tables

**Table 1:** Comparison of characteristics between Bobath and conventional medical gymnastics groups.

CATEGORY		BOBATH THERAPY (*n* = 50)			CONVENTIONAL THERAPY (*n* = 50)			*p*-Value *
		N	%		N	%		
SEX	Male	25	50.0%		26	52.0%		0.841
	Female	25	50.0%		24	48.0%		
APGAR 1st min	4	0	0.0%		1	2.0%		0.714
	5	1	2.0%		1	2.0%		
	9	7	14.0%		10	20.0%		
	10	42	84.0%		38	76.0%		
NUMBER OF RISK FACTORS	0	19	38.0%		17	34.0%		0.939
	1	23	46.0%		26	52.0%		
	2	6	12.0%		5	10.0%		
	3	2	4.0%		2	4.0%		
TWIN	No	41	82.0%		42	84.0%		>0.999
	Yes	9	18.0%		8	16.0%		
PREMATURE	No	48	96.0%		48	96.0%		>0.999
	Yes	2	4.0%		2	4.0%		
HYPOTHYROIDISM	No	33	66.0%		35	70.0%		0.830
	Yes	17	34.0%		15	30.0%		
GESTATIONAL DIABETES	No	44	88.0%		42	84.0%		0.774
	Yes	6	12.0%		8	16.0%		
INTRAUTERINE GROWTH RESTRICTION	No	46	92.0%		44	88.0%		0.741
	Yes	4	8.0%		6	12.0%		
HASHIMOTO DISEASE	No	49	98.0%		49	98.0%		>0.999
	Yes	1	2.0%		1	2.0%		
COLESTASIS	No	49	98.0%		50	100.0%		>0.999
	Yes	1	2.0%		0	0.0%		
HYPERTENSION	No	48	96.0%		48	96.0%		>0.999
	Yes	2	4.0%		2	4.0%		
REANIMATION	No	50	100.0%		49	98.0%		>0.999
	Yes	0	0.0%		1	2.0%		
PLACENTA PREVIA	No	50	100.0%		49	98.0%		>0.999
	Yes	0	0.0%		1	2.0%		
MODE OF BIRTH	Vaginal	33	66.0%		27	54.0%		0.307
	C-section	17	34.0%		23	46.0%		
BRAIN ULTRASOUND FINDINGS	1st degree	42	84.0%		38	76.0%		0.454
	2nd degree	8	16.0%		12	24.0%		
		**Median**	**Q1**	**Q3**	**Median**	**Q1**	**Q3**	*p*-value **
AGE OF MOTHER (years)		32.00	28.00	36.00	33.00	28.00	36.00	0.658
BIRTH WEIGHT (kg)		3.270	2.673	3.483	3.020	2.403	3.343	0.072
BIRTH LENGTH (cm)		48.00	45.75	50.00	47.00	44.00	49.25	0.420
GESTATIONAL AGE (days)		273.00	260.75	280.00	266.50	257.75	280.00	0.491
AGE OF INCLUSION (days)		100.00	92.75	112.00	105.50	96.75	119.75	0.073

* Fisher’s exact test; ** Mann–Whitney U test.

**Table 2:** Comparison of communication skills, problem-solving skills, and psycho-social status dynamics (Ages and Stages questionnaire).

CATEGORY (AGE)	BOBATH THERAPY (*n* = 50)			CONVENTIONAL THERAPY (*n* = 50)			*p*-Value *
	N_OS_ (%)	N_MON_ (%)	N_NOS_ (%)	N_OS_ (%)	N_MON_ (%)	N_NOS_ (%)	
COMMUNICATION SKILLS (3 MONTHS)	0.0	26.0	74.0	0.0	26.0	74.0	>0.999
COMMUNICATION SKILLS (6 MONTHS)	1.0	80.0	6.0	18.0	70.0	12.0	0.437
COMMUNICATION SKILLS (9 MONTHS)	76.0	24.9	0.0	78.0	22.0	0.0	>0.999
PROBLEM-SOLVING SKILLS (3 MONTHS)	2.0	26.9	72.0	0.0	28.0	72.0	>0.999
PROBLEM-SOLVING SKILLS (6 MONTHS)	18.0	76.0	6.0	20.0	74.0	6.0	>0.999
PROBLEM-SOLVING SKILLS (9 MONTHS)	84.0	16.0	0.0	84.0	16.0	0.0	>0.999
PSYCHO-SOCIAL STATUS (3 MONTHS)	2.0	34.0	64.0	4.0	32.0	64.0	>0.999
PSYCHO-SOCIAL STATUS (6 MONTHS)	34.0	66.0	0.0	32.0	66.0	2.0	>0.999
PSYCHO-SOCIAL STATUS (9 MONTHS)	92.0	8.0	0.0	92.0	8.0	0.0	>0.999

* Fisher’s exact test; N_XX_ (%)—percentage of participants per category; OS—on schedule; MON—monitor; NOS—not on schedule.

**Table 3:** Subjective evaluation of progress in social contact and interactions via questionnaire filled out by legal guardians.

QUESTION	ANSWERS	BOBATH THERAPY (*n* = 50)		CONVENTIONAL THERAPY (*n* = 50)		*p*-Value *
		N	N (%)	N	N (%)	
IS THERE A NOTICEABLE IMPROVEMENT IN SOCIAL CONTACT AND INTERACTIONS?	(a) Yes	47	94.0	47	94.0	>0.999
	(b) No	3	6.0	3	6.0	
IF THERE IS A NOTICEABLE IMPROVEMENT, WOULD YOU ATTRIBUTE IT TO THE THERAPY?	(a) Yes, I attribute it to daily therapeutic sessions	28	59.6	27	57.4	>0.999
	(b) No, I would attribute it to my child’s course of development	19	40.4	20	42.6	

* Fisher’s exact test; N—absolute number of participants per category; N (%)—percentage of participants per category.

**Table 4:** Comparison of activity log characteristics.

CATEGORY	BOBATH THERAPY (*n* = 50)			CONVENTIONAL THERAPY (*n* = 50)			*p*-Value *
	Median	Q1	Q3	Median	Q1	Q3	
NUMBER OF DAYS EXERCISED	159.00	140.00	170.00	166.50	155.75	175.50	0.012
NUMBER OF WEEKEND DAYS EXERCISED	39.50	32.00	45.25	41.50	36.75	46.00	0.148
LENGTH OF SINGLE SESSION (min)	30.00	30.00	35.00	30.00	30.00	31.25	0.422
NUMBER OF DAILY SESSIONS	3.00	3.00	4.00	3.00	3.00	4.00	0.755
NUMBER OF DAYS TO FIRST NOTICEABLE IMPROVEMENT	30.00	20.00	45.00	30.00	25.00	45.00	0.447

* Mann–Whitney U test, 0.0xx—statistically significant *p*-value.

**Table 5:** Longitudinal comparison of Munich Functional Developmental Diagnostic Scale results between examinations at 3 months, 6 months, and 9 months of age.

CATEGORY	BOBATH THERAPY		CONVENTIONAL THERAPY	
	Test Value *	*p*-Value *	Test Value *	*p*-Value *
PERCEPTION AGE DELAY	100.000	<0.001	100.000	<0.001
SPEECH AGE DELAY	100.000	<0.001	100.000	<0.001
SOCIALIZATION AGE DELAY	99.508	<0.001	100.000	<0.001

* Friedman test, 0.0xx—statistically significant *p*-value.

**Table 6:** Longitudinal comparison of Munich Functional Developmental Diagnostic Scale results between examinations at 3 and 6 months, 6 and 9 months, and 3 and 9 months of age.

CATEGORY	BOBATH THERAPY		CONVENTIONAL THERAPY	
	Comparison	*p*-Value *	Comparison	*p*-Value *
PERCEPTION AGE DELAY (days)	3 months vs. 6 months	<0.001	3 months vs. 6 months	<0.001
	6 months vs. 9 months	<0.001	6 months vs. 9 months	<0.001
	3 months vs. 9 months	<0.001	3 months vs. 9 months	<0.001
SPEECH AGE DELAY (days)	3 months vs. 6 months	<0.001	3 months vs. 6 months	<0.001
	6 months vs. 9 months	<0.001	6 months vs. 9 months	<0.001
	3 months vs. 9 months	<0.001	3 months vs. 9 months	<0.001
SOCIALIZATION AGE DELAY (days)	3 months vs. 6 months	<0.001	3 months vs. 6 months	<0.001
	6 months vs. 9 months	<0.001	6 months vs. 9 months	<0.001
	3 months vs. 9 months	<0.001	3 months vs. 9 months	<0.001

* Wilcoxon signed-rank test, 0.0xx—statistically significant *p*-value.

**Table 7:** Cross-sectional comparison of Munich Functional Developmental Diagnostic Scale results between cohorts.

CATEGORY	AGE	BOBATH THERAPY (*n* = 50)			CONVENTIONAL THERAPY (*n* = 50)			*p*-Value *
		Median	Q1	Q3	Median	Q1	Q3	
PERCEPTION AGE DELAY (days)	3 MONTHS	20.50	17.00	22.00	22.00	17.75	24.00	0.111
	6 MONTHS	14.00	11.00	15.25	14.00	11.75	16.00	0.289
	9 MONTHS	6.00	4.00	7.00	6.50	5.00	8.00	0.201
SPEECH AGE DELAY (days)	3 MONTHS	19.50	17.00	21.00	22.00	18.50	24.00	0.002
	6 MONTHS	11.00	9.75	15.00	13.50	10.75	15.25	0.030
	9 MONTHS	4.50	2.75	6.00	5.00	2.00	7.25	0.440
SOCIALIZATION AGE DELAY (days)	3 MONTHS	19.00	17.00	22.00	22.00	19.50	24.25	0.019
	6 MONTHS	11.00	8.75	14.25	12.00	10.00	16.00	0.152
	9 MONTHS	3.00	1.00	6.00	5.00	2.00	6.25	0.042

* Mann–Whitney U test; Q1—1st quartile; Q3—3rd quartile; 0.0xx—statistically significant p-value.

## Data Availability

All the research data are included in the manuscript.
